# Physicochemical properties and survival assessment of potential probiotics in a novel dairy drink during storage

**DOI:** 10.1002/fsn3.3697

**Published:** 2023-10-26

**Authors:** Oğuzhan Gedik, Aynur Gül Karahan

**Affiliations:** ^1^ Department of Food Engineering, Faculty of Engineering Süleyman Demirel University Isparta Turkey

**Keywords:** dairy drink, fermented milk, lactic acid bacteria, probiotics, yeasts

## Abstract

A novel fermented dairy drink utilizing microbial strains displaying potential probiotic attributes was formulated. The study constituted several experimental cohorts, including *Lactiplantibacillus plantarum* AB6‐25, isolated from a human stool sample; *Lacticaseibacillus casei* K2, sourced from a koumiss sample; *Lacticaseibacillus rhamnosus* 3B7, derived from a traditional yogurt sample; and identical lactic acid bacteria (LAB) in combination with yeast (*Saccharomyces boulardii* T8‐3C from chicken feces) strains. Two distinct iterations of probiotic‐fermented dairy drinks were generated by introducing subcultured microorganism cultures: LAB strains at a concentration of 1% each (designated as combination A) and a blend of LAB strains at 1% each along with T8‐3C at 0.5% (designated as combination B) into both whole and semi‐skimmed milk matrices. The fermentation process persisted until the pH reached 4.6 under constant conditions of 37 ± 1°C. Subsequently, the samples were held at 4 ± 1°C for 15 days. The groups' physicochemical, microbiological, and sensory characteristics were determined on days 1, 8, and 15 of storage, and the protein profile was determined. Standardized regression analysis and principal component analysis evaluated the results. Fat content affected the changes in dry matter. pH decreased in all samples during storage, particularly in the yeast group. The microorganism group positively affected syneresis, whereas fat content and the interaction of fat content and the microorganism group had a negative effect. The most critical factor in the decrease in syneresis was the increase in fat content. LAB and yeasts maintained their probiotic effects during storage, with a viability level of approximately 10^9^ and 10^8^ cfu/mL, respectively.

## Graphical abstract

A new fermented dairy drink was developed using microbial strains with potential probiotic properties. Experimental groups were formed by inoculating lactic acid bacteria (LAB) and LAB+yeast strains into whole and semi‐skimmed milk. The groups' physicochemical, microbiological, and sensory characteristics were determined on days 1, 8, and 15 of storage, and the protein profile was determined. Standardized regression analysis and principal component analysis evaluated the results.

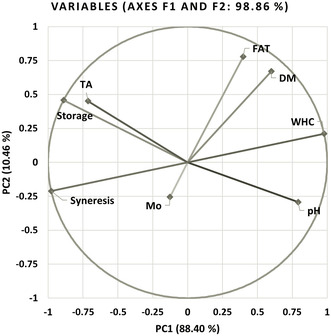

## 1 INTRODUCTION

Functional foods positively affect consumer health when consumed in sufficient quantities (Khorshidian et al., 2020; Kılıç et al., 2009). Probiotic foods constitute many functional foods (Mishra & Mishra, 2012). The Food and Agriculture Organization of the United Nations/World Health Organization (FAO/WHO) defines probiotics as “live microorganisms that are beneficial to the health of the host when taken in sufficient quantities” (FAO/WHO, 2002). Also, the statement “this food contains probiotics” indicates that food must contain at least 10^6^–10^7^ cfu/g probiotic microorganisms throughout its shelf life (Bambace et al., 2021).

The most commonly used microorganisms as probiotics are lactic acid bacteria (LAB), such as *Lactobacillus* spp., *Bifidobacterium* spp., *Streptococcus* spp., and *Enterococcus* spp. These are present in the gut microbiota of humans and warm‐blooded animals (Abdel‐Hamid et al., 2019; Kahraman & Karahan, 2018; Khorshidian et al., 2020) and traditionally fermented foods and drinks (Kiani, Nami, Hedayati, Elieh Ali Komi, et al., 2021; Kiani, Nami, Hedayati, Jaymand, et al., 2021). Apart from LAB, *Bacillus* spp., yeasts (*S. cerevisiae* and *S. boulardii*), and filamentous fungi (*Aspergillus oryzae*) are also used in probiotic preparations (Parvez et al., 2006).

The beneficial effects of probiotics on health are thought to take place in three ways. They inhibit pathogenic and harmful bacteria in the intestinal tract, regulate microbial metabolism (enzymatic activity), and positively affect the immune system (Fuller, 1991; Kahraman & Karahan, 2018).

There has been considerable attention toward LAB due to their capacity to modulate the human host system's response to foodborne pathogens. As a result, there is a current exploration of these bacteria's potential employment as a bio‐preservative agent within the food and dairy sectors, and as an alternative to antibiotics in both human and animal medical treatments and animal breeding initiatives (Boranbayeva et al., 2020; Kahieshesfandiari et al., 2021; Khushboo et al., 2023; Nami et al., 2016, 2018, 2022).

LAB represent a noteworthy category of bacteria extensively employed in the food, dairy, probiotic, and drink manufacturing industries. Notably acknowledged as generally regarded as safe (GRAS), these bacteria exhibit distinctive attributes that render them exceptionally suitable for the abovementioned applications. In the context of the food sector, especially concerning the production of foundational or starter cultures for diverse dairy products, LAB, particularly *Lactobacillus* spp., find widespread employment (Khushboo et al., 2023). The products obtained from the fermentation of milk with probiotic microorganisms are called “probiotic dairy products.” The main probiotic dairy products are yogurt, fermented milk, and cheese (De Andrade et al., 2019; Kılıç et al., 2009). Fermented dairy drinks can be produced traditionally or industrially to meet the needs of larger masses. Examples of traditional and industrial fermented dairy drinks include acidophilus yogurt and milk in Scandinavian countries, Cultura in Denmark, Biogarde and Bifidus‐milk in Germany, Aco‐yogurt in Switzerland, Gerilac in Finland, Ofilus in France, Yakult in Japan, and Kefir and Koumiss in the Caucasus (Turkmen et al., 2019). Particularly, within the realm of functional foods, dairy‐based drinks currently exhibit notable activity. This is attributed to several factors: (i) their capacity to be enriched with specific nutrients and bioactive components, (ii) their convenience in addressing diverse consumer needs, and (iii) their ease of distribution and adaptable shelf‐stability under preferable refrigerated storage conditions (Kausar et al., 2012). The assortment of commercially accessible products can be classified as follows: (1) dairy‐based drinks fortified with probiotic microorganisms and diverse minerals, as well as ω‐3 enriched drinks, (2) plant‐based and fruit‐derived drinks, and (3) drinks designed to boost energy. Functional dairy drinks can be divided into three main categories: those with added nutrients, those based on whey, and those originating from whey with nutraceutical properties (Hati et al., 2019).

The global functional dairy drinks market is growing daily due to their health importance. Apart from traditional dairy drinks such as kefir, drinking yogurt, and milk, the current valuation of the dairy‐based drinks market is US$73.77 billion (Skyquest, 2023).

In addition to the dairy‐based functional products containing probiotics sold on the market, various studies have been conducted to develop new products. In some of these studies, different milk components such as whey and different probiotics were used to develop products (Saglam et al., 2019; Skryplonek et al., 2019), while in others, various fruit juices, concentrates, and/or pulps and cereals were added to whey and fermented with probiotics. In some studies, milk is also included in these mixtures (De Andrade et al., 2019). Using fruit products and cereals in addition to milk and whey in the composition, the aim was to strengthen the health effect and utilize the protective effect of the components on the viability of probiotics during the storage period.

In the present study, the microorganisms used to produce functional fermented dairy drinks were isolated from different sources in various previous studies. *Lactiplantibacillus plantarum* AB6‐25 was isolated from the fecal samples of healthy individuals. Tolerance to acidic conditions and bile salts, antibiotic susceptibility, and other probiotic properties of the bacteria were determined, and genotypic identification was carried out (Başyiğit, 2004; Kiliç & Karahan, 2010). Also, it was determined that it maintained its viability at 30%–35% in the TIM‐1 system (Yıldıran Yılmaz et al., 2013). *Lacticaseibacillus casei* K2 was isolated from a Kazakhstan‐specific koumiss sample, its probiotic properties (tolerance to acidic conditions and bile salts, antibiotic susceptibility, and antagonistic activity against some pathogenic bacteria) were determined, and genotypic identification was carried out (Boranbayeva et al., 2020). *Lacticaseibacillus rhamnosus* 3B7 isolated from traditional yogurt samples has superior technological properties such as rapid acid production and exopolysaccharide formation, and it has been phenotypically identified (Çetin, 2017).

The high yeast levels found in kefir grains and fermented dairy products represent a distinctive characteristic of conventional fermented dairy commodities. The majority of probiotic microorganisms available on the market consist of bacteria, notably lactobacilli and bifidobacteria. Other strains, for instance, *S. boulardii* (Bourrie et al., 2016), have demonstrated potential in ameliorating symptoms related to *Clostridium difficile*‐induced diarrhea, mitigating inflammation, and inducing alterations in intestinal immune responses and conditions. Furthermore, the yeast strain *S. boulardii* has been recognized as a probiotic agent capable of mitigating diarrhea triggered by antibiotic usage (García‐Burgos et al., 2020). Consequently, *S. boulardii* was also encompassed within the scope of this research. The specific *S. boulardii* T8‐3C employed in this investigation was isolated from avian fecal samples. This strain exhibited notably enhanced probiotic attributes such as survival in gastric juice, aggregation and coaggregation, and EPS production and underwent genotypic characterization (Yıldıran et al., 2019).

The present study prepared two probiotic mixtures using three different LAB and one yeast strain. It has aimed to obtain a novel product by combining the different probiotic properties of LAB and/or yeast strains. These two probiotic culture mixtures were inoculated into whole and semi‐skimmed milk to produce probiotic‐fermented dairy drinks. The physicochemical and microbiological properties of the samples were determined weekly during the 15‐day storage period. Also, protein profiles by SDS‐PAGE and the sensory properties of the samples were determined. Regression analysis and principal component analysis (PCA) were performed to reveal the relationship between explanatory and response variables. Multivariate linear regression analysis was applied to the experimental data to reveal the effect of independent variables on the dependent variables.

## 2 MATERIALS AND METHODS

### 2.1 Materials

The probiotic milk drinks were produced using commercial whole (3% fat) and semi‐skimmed (1.5% fat) UHT milk. Two different culture combinations were produced with microorganisms. Combination A contained *L. plantarum* AB6‐25 (GenBank Accession No. GQ332649), *L. rhamnosus* 3B7, and *L. casei* K2 (GenBank Accession No. MK643164), while combination B included *S. boulardii* T8‐3C (GenBank Accession No. MG711551) in addition to these strains.

### 2.2 Methods

#### 2.2.1 Production of microorganisms

The stock cultures of LAB maintained at −20°C in De Man, Rogosa, and Sharp (MRS) broth (Merck, Darmstadt, Germany) containing 20% glycerol were incubated for 18 h at 37°C using MRS broth. The stock cultures of *S. boulardii* T8‐3C maintained at −20°C in malt extract–yeast extract–peptone–glucose broth (MYPG) containing 20% glycerol were incubated for 18 h at 30°C using MYPG (Syal & Vohra, 2013). Stock cultures were subcultured twice.

#### 2.2.2 Probiotic fermented milk drink production

After the temperature of the milk samples was raised to 37°C, two different probiotic‐fermented dairy drinks were produced by inoculating subcultured microorganism cultures LAB (1% each strain, combination A) and LAB (1% each strain) + T8‐3C (0.5%, combination B) into commercial whole and semi‐skimmed UHT milk samples. The samples were incubated at 37 ± 1°C. Fermentation was terminated when the pH values of the probiotic‐fermented milk drink samples reached 4.6. The samples were then stored at 4 ± 1°C for 15 days. The products' physicochemical, microbiological, and sensory properties were determined on days 1, 8, and 15 of storage. In addition, the protein profile was analyzed by SDS‐PAGE on days 1 and 15 of storage. The experiments were carried out in two replicates.

#### 2.2.3 Physicochemical and sensory analyses

The percentages of DM, fat, and titratable acidity were determined according to the Turkish Standard 591 (Öner & Şanlıdere Aloğlu, 2018). The pH values were measured using a digital pH meter (Seven Compact™ S220, Mettler Toledo, Columbus, OH, USA). The L*, a*, b*, *C*, and *H*° values were determined with a colorimeter (PCE‐TCR 200, Hong Kong, China). Syneresis and water holding capacity were performed according to Hussain et al. (2016). In addition to these analyses, sensory analyses were performed using the scoring method and parameters recommended in the TS 1330 Yogurt Standard (TSE, 2006).

#### 2.2.4 Microbiological analyses

Samples were taken from the groups under aseptic conditions, and dilutions were prepared in sterile physiological saline (PS). Appropriate dilutions were inoculated onto MRS agar for LAB enumeration and onto MYPG agar for yeast enumeration by the drop culture method (Başyiǧit et al., 2006; Yıldıran et al., 2019). The plates were incubated at 37°C for 48 h and then enumerated.

#### 2.2.5 Sodium dodecyl sulfate polyacrylamide gel electrophoresis (SDS‐PAGE)

A 50 mg sample of fermented milk containing probiotics was taken, and 400 μL of loading buffer was added and mixed. The samples were kept in a hot water bath at 95°C for 5 min. The SDS‐PAGE analysis was performed using a Mini Protean II (Bio‐Rad Lab Inc., Hercules, CA, USA) device according to the method Laemmli (1970) recommended. Proteins were visualized with a Coomassie Brilliant Blue stain. Molecular weights of the proteins were calculated in GelAnalyzer 19.1 (www.gelanalyzer.com by Istvan Lazar Sr., Ph.D., CSc), using a standard protein marker (Wide MW Range, M 4038 Sigma, Burlington, MA, USA).

#### 2.2.6 Statistical analysis

The microbiological, physicochemical, and sensory analyses of probiotic‐fermented milk drinks were statistically evaluated by analysis of variance. Regression and PCA were applied to reveal the relationship between explanatory and response variables. Multivariate linear regression analysis was applied to the experimental data to reveal the effect of independent variables on the dependent variables. Regression coefficients (RC) were calculated for direct effect and binary interaction terms. RC values can be used to determine the magnitude and direction of the effect of an explanatory variable on model prediction. However, standardization of regression coefficients is generally recommended to compare the relative effects of independent variables due to the different variable units and ranges (Borgonovo & Plischke, 2016; Hamby, 1994). Therefore, standardized regression coefficients (SRCs) were calculated for the selected model parameters and ranked according to their impact on model estimation. PCA variable plots were utilized to explain the relationships between the variables. Before applying PCA, centralization (subtracting the mean value from each data column) and scaling (dividing the centralized data column by the standard deviation) were applied to the data. Using the R language, all statistical calculations were performed with RStudio (version 1.1.383; RStudio, Boston, MA, USA). Multivariate linear regression analysis was performed utilizing the “lm” function. These commands are available in the “stat” package (R Core Team, 2023). Before the regression analysis, the microorganism group, a categorical variable, was coded as −1 and +1 for microorganisms A and B, respectively. For the standardization of RC values, the “lm.beta” (Behrendt, 2015) command was used in the “lm.beta” package. PCA analysis and related graphs were generated using the “factoextra” package (Kassambara & Mundt, 2016).

## 3 RESULTS AND DISCUSSION

### 3.1 Physicochemical properties

**FIGURE 1:**
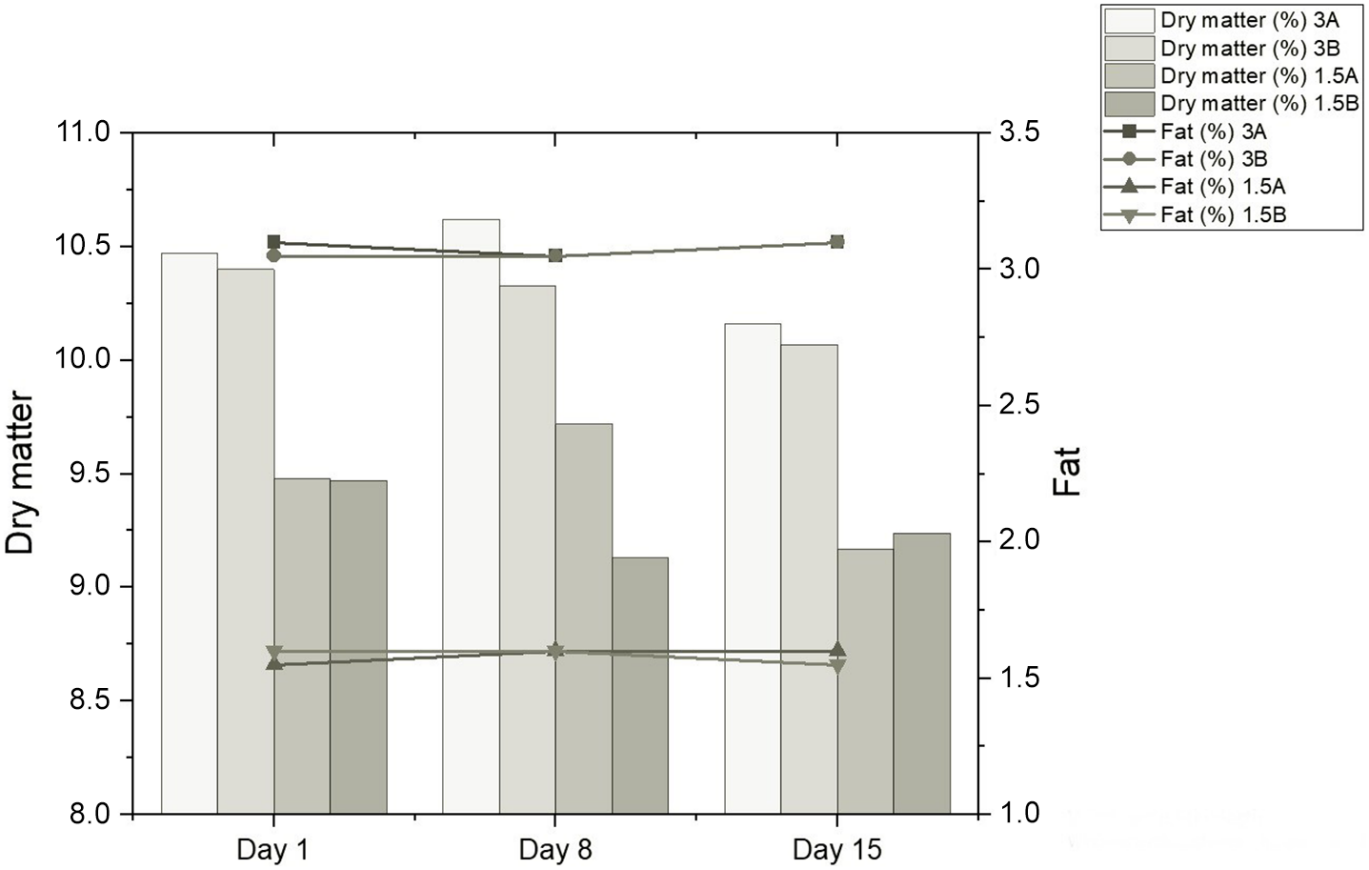
Changes in the dry matter and fat content of fermented dairy drinks during storage (%).

The dry matter of whole and semi‐skimmed UHT milk used in the production of probiotic‐fermented dairy drinks was determined to be 11.42 ± 0.12 and 10.2 ± 0.14, pH as 6.58 ± 0.03 and 6.59 ± 0.02, and acidity as 0.140% ± 0.005 and 0.144% ± 0.005 lactic acid, respectively. Fermented dairy drinks were produced using UHT milk and probiotic–microorganism combinations. Physicochemical analyses were carried out using the samples taken on fermentation days 1, 8, and 15. The changes in dry matter and fat content during storage are given in Figure 1.

**FIGURE 2:**
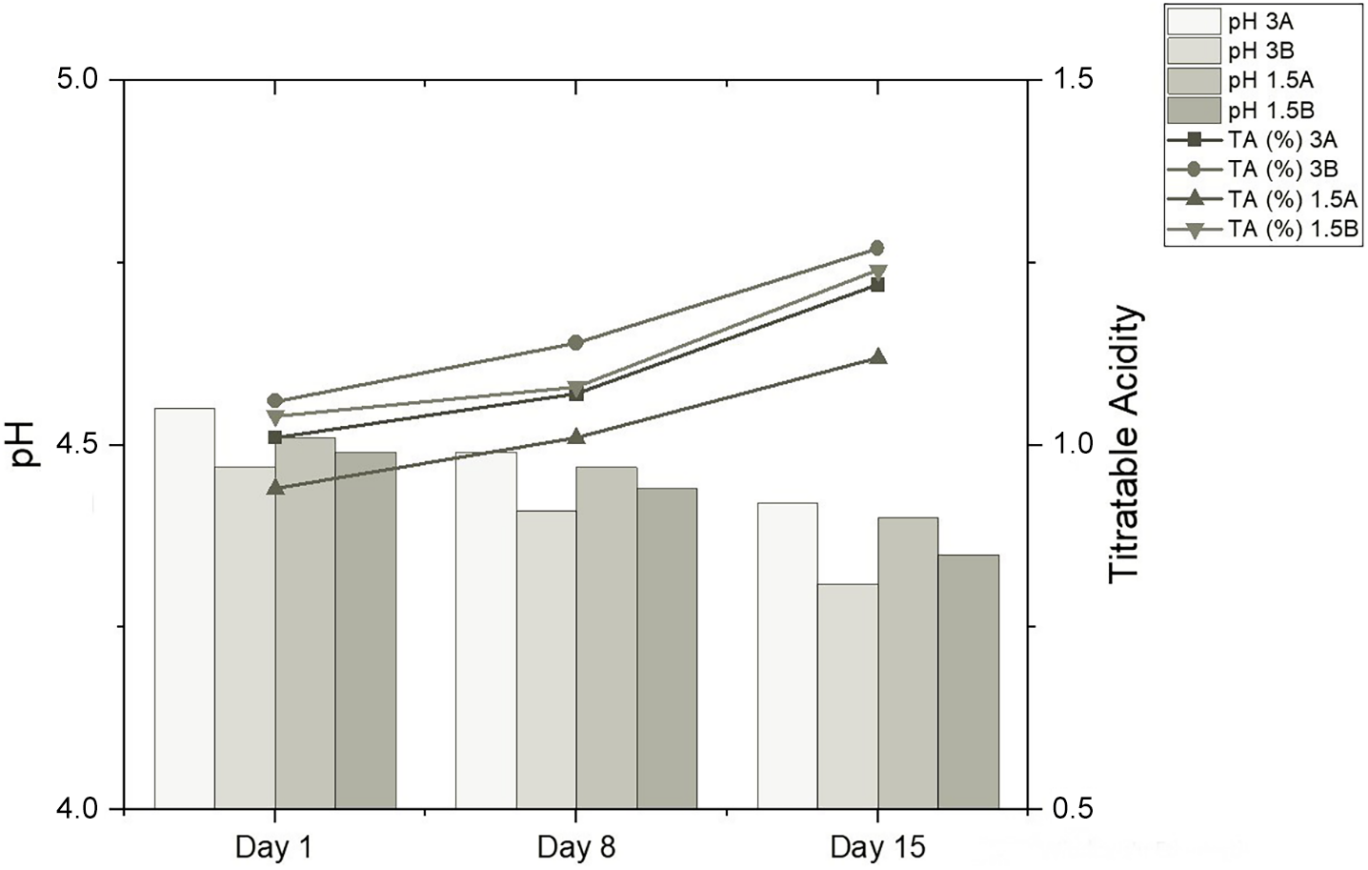
Changes in the pH and titratable acidity during storage of probiotic‐fermented dairy drinks (%).

Accordingly, the fat content was found to be statistically significant for the change in dry matter. The effect of this parameter was found to be positive. The dry matter content is expected to increase with the increase in fat content. In addition to fat, other components added to milk also increase for dry matter. Skryplonek et al. (2019) produced various drinks using different concentrations of whey, milk, condensed milk, skim milk powder, and probiotic cultures. Like in the present study, dry matter increased with increasing concentrations of drink ingredients. Figure 2 shows the changes in pH and acidity during storage.

It was determined that the pH value of the probiotic‐fermented milk drink was affected by both the interaction of the microorganism group and fat content (*p* < .05) and storage time (*p* < .05). Also, this effect was found to be negative. The pH value decreased in the subsequent days of storage. Group A had higher pH values than group B. However, the actual effects of the microorganisms used vary within a minimal range. Potential probiotic cultures used in the production of probiotic dairy drinks have a significant effect on the pH of the drinks. Different genera, species, and even strains of LAB in the probiotic culture affect the changes in pH. For example, Drgalic et al. (2005) have reported that the pH values of the drinks produced with *L. casei* Lc‐01 and *Lactobacillus acidophilus* La‐5 decreased after 28 days of storage. In contrast, the pH value of the drink produced with  *Bifidobacterium bifidum* Bb‐12 did not significantly change.

In the present study, the only difference between groups A and B regarding microorganisms was using *S. boulardii* T8‐3C in group B. Therefore, it was concluded that T8‐3C caused the decrease in pH in group B. Some *S. boulardii* strains are known to produce acetic acid. Offei et al. (2019) suggested that the high concentration of acetic acid produced by some *S. boulardii* strains is responsible for the antibacterial effect, and explained the genetic basis of this property.

Storage time was the most critical parameter affecting lactic acid concentration (*p* = .052). The relationship between these two parameters was found to be positive. Similarly, Skryplonek et al. (2019) reported that the lactic acid concentration in probiotic‐fermented milk drinks produced with *L. acidophilus* LA‐5 and  *Bifidobacterium animalis* subsp. *lactis* BB‐12 increased slightly during storage.

**FIGURE 3:**
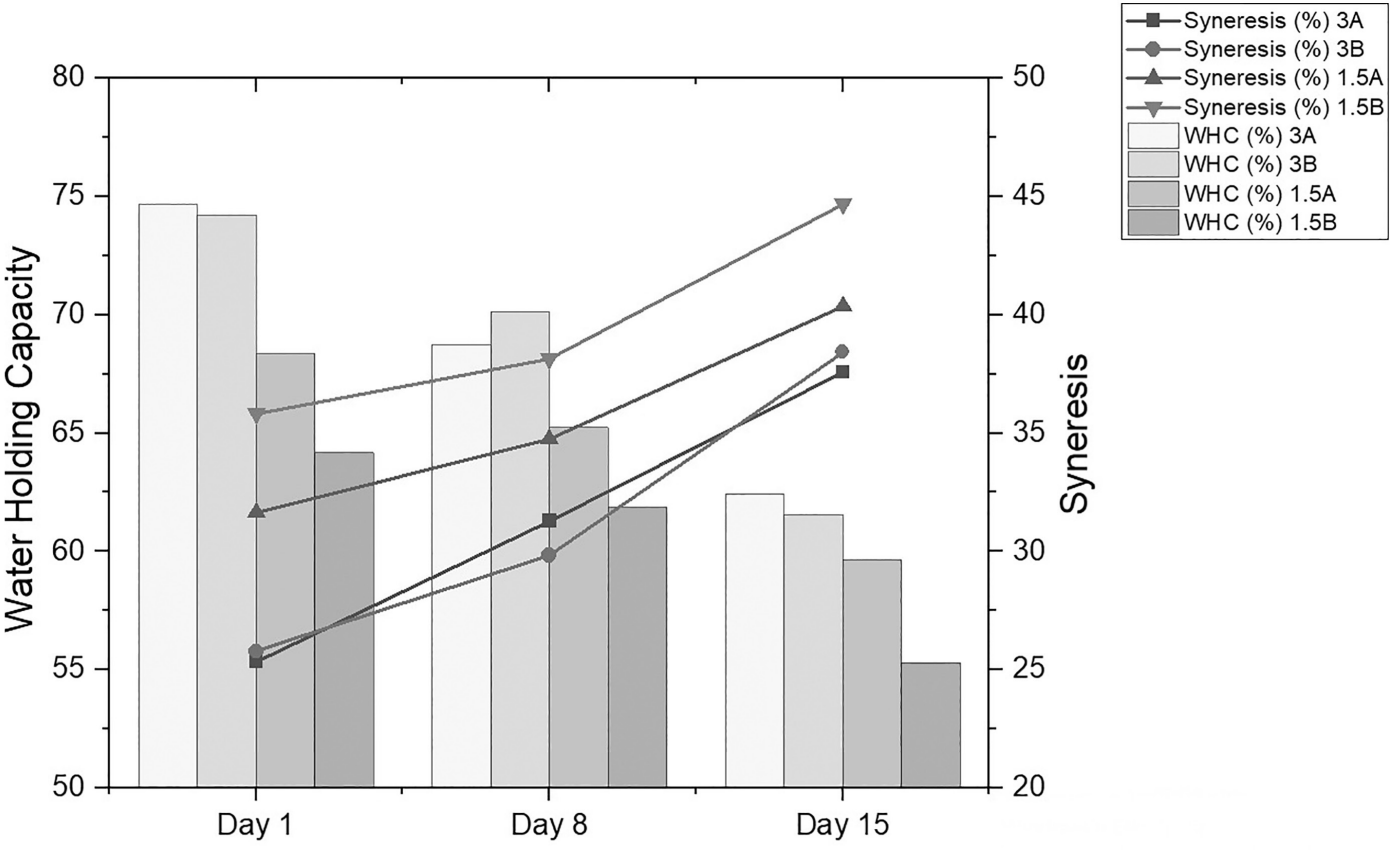
Changes in the syneresis and water holding capacity of probiotic‐fermented dairy drinks during storage (%).

The syneresis and water‐holding data of fermented milk drinks produced with potential probiotic strains are given in Figure 3.

Syneresis stands as a crucial factor concerning the textural attributes of fermented dairy products and holds notable importance in terms of both stability and consumer reception. Among diverse procedural factors like heat treatment, fermentation, storage circumstances, and starter culture, the composition of the milk base notably influences the formation of gel structure (El Bouchikhi et al., 2019). Consequently, in a descending hierarchy of significance, the content of fat (with a significance level of *p* < .01), the type of microorganism group, and the interplay between microorganism group and fat content exhibited statistically noteworthy effects (with a significance level of *p* < .05) on alterations in the syneresis‐dependent variable. Among these parameters, the microorganism group was found to be positively effective, whereas fat content and the interaction of the microorganism group and fat content were negatively effective. Increasing milk fat levels reduces the syneresis rate (Mateo et al., 2009). Fat globules obstruct the outward movement of moisture from the curd (McSweeney, 2007). Also, EPS‐producing starter cultures are important in reducing or eliminating syneresis (Amatayakul et al., 2006). *L. rhamnosus* 3B7 and *S. boulardii* T8‐3C, used in the present study, are strains that produce exopolysaccharides (Çetin, 2017; Yıldıran et al., 2019). Therefore, it was thought that they might be effective in decreasing syneresis. The beneficial effects of extracellular polymeric substance (EPS) production on human health have been substantiated through various investigations. Scientific inquiry has revealed that EPS synthesized by probiotic microorganisms exhibits various physiological abilities. These encompass a range of beneficial properties such as antioxidative, immune‐modulatory, antitumor, anti‐inflammatory, antiviral, and hypotensive characteristics, as documented by researchers (Araya et al., 2002; Kahraman et al., 2022). In contrast to undergoing gastrointestinal tract degradation processes, EPS reaches the cecum and colon without being catabolized. Within these regions, they undergo fermentation by the gut microbiota. This fermentation process yields advantageous byproducts, notably short‐chain fatty acids (SCFA). This dual action leads to a simultaneous decrease in pH levels and the suppression of pathogenic proliferation. Additionally, this process enhances the prevalence of beneficial bacteria, provides a source of energy for colonic epithelial cells, and fortifies the integrity of the intestinal barrier, as supported by recent research (Ma et al., 2021). Furthermore, EPS plays a crucial role in safeguarding against various challenges. These include desiccation, phagocytosis, cellular recognition, phage attacks, the effects of antibiotics or toxic compounds, and osmotic stress, as highlighted in the work of Angelin and Kavitha (2020).

The color values of foods are essential parameters in terms of consumer appreciation. Therefore, the change in color values during the storage process was monitored, and the results are given in Table 1.

**TABLE 1:** Change in color values of probiotic‐fermented milk beverages during storage.

Color values	Storage (day)	Groups			
		3A^a^	3B	1.5A	1.5B
*L* *	1	22.98 ± 0.49	24.78 ± 1.02	24.29 ± 0.49	22.91 ± 1.11
	15	20.32 ± 0.21	19.97 ± 1.25	23.46 ± 0.70	21.45 ± 0.74
*a* *	1	−5.75 ± 0.81	−5.54 ± 0.43	−5.67 ± 0.32	−5.79 ± 1.03
	15	−5.77 ± 0.09	−5.81 ± 0.98	−5.66 ± 0.19	−5.45 ± 0.46
*b* *	1	−16.17 ± 10.58	−9.67 ± 2.49	−8.01 ± 0.67	−15.90 ± 11.04
	15	6.20 ± 2.12	2.84 ± 3.37	4.02 ± 0.61	3.72 ± 0.43
*C*	1	17.48 ± 9.52	11.20 ± 1.94	9.82 ± 0.36	11.41 ± 2.64
	15	8.68 ± 1.49	6.94 ± 0.67	6.68 ± 0.26	6.25 ± 0.49
*H*°	1	245.80 ± 16.70	239.49 ± 8.38	234.63 ± 3.82	239.30 ± 1.82
	15	133.95 ± 11.07	165.6 ± 40.80	152.69 ± 2.57	156.06 ± 4.87

^a^ 3A, LAB into whole UHT milk; 3B, LAB+T8‐3C into whole UHT milk; 1.5A, LAB into semi‐skimmed UHT milk; 1.5B, LAB+T8‐3C into semi‐skimmed UHT milk.

During the storage process, lightness (L*) values slightly decreased, redness (a*) values remained unchanged, yellowness (b*) values increased in all groups, especially in group A produced from whole milk, and chroma (*C*) and hue (*H*°) values decreased in all groups. The color of fermented drinks is frequently linked to the existence of natural dyes within the initial ingredients. Furthermore, alterations in both storage duration and pH levels might influence the chromatic attributes of fermented foods (dos Santos et al., 2019; Łopusiewicz et al., 2019). Accordingly, the variables that significantly affected the L* color value were determined as the binary interaction of the microorganism group and storage time and microorganism group in decreasing order of importance. The effect of statistically significant parameters was negative, whereas that of non‐significant parameters was positive. A slight decrease in L* value can be accepted as usual since microorganisms may cause proteolysis and a consequent decrease in L* value during the storage process in probiotic‐fermented drinks (Costa et al., 2017). There are no significant effects of any variable on a* color value. Therefore, the model constant (intercept) is statistically significant and equal to the mean of all observations. The variables with a statistically significant effect on b* color value are, in descending order of importance, the interaction of the microorganism group and fat concentration (positive direction of relationship), microorganism group (negative direction of relationship), the binary interaction of microorganism group and storage time (positive direction of relationship) and the direct effect of storage time (positive direction of relationship). The results partially agreed with the studies of Randazzo et al. (2016) and Łopusiewicz et al. (2019), who noted a reduction in lightness with increased b* values during storage. However, dos Santos et al. (2019) observed significantly increased lightness in fermented soymilk drinks. Ryan et al. (2020) reported a significant change (*p* > .05) in the a* value throughout the storage of fermented probiotic drinks containing mango juice. The only effective parameter for *C* and *H*° values is the storage time. Both values decreased during storage. Similarly, dos Santos et al. (2019) determined that both values were significantly affected by the storage period in soy milk drinks fermented using kefir grains.

### 3.2 Microbiological properties

**FIGURE 4:**
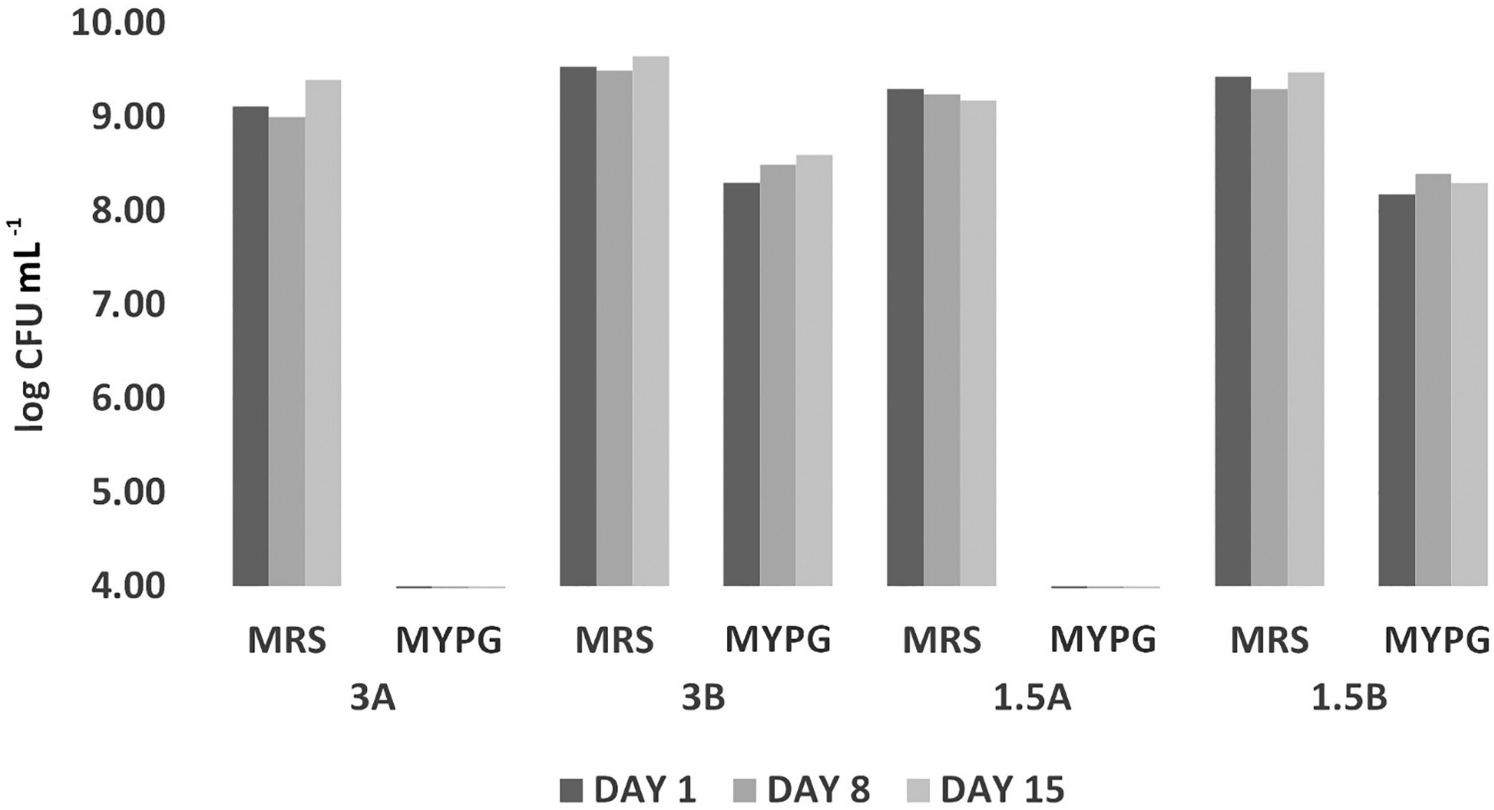
Changes in the microbiological properties of fermented dairy drinks during storage (log_10_ CFU/g).

The changes in LAB and yeast counts of probiotic‐fermented dairy drinks during the storage period are given in Figure 4.

One of the most important factors when considering the quality of probiotic products is the count of viable probiotic bacteria cells. The probiotic bacteria count should be at least 6 log cfu/mL during storage until expiration (Skryplonek et al., 2019). The counts of both groups of potential probiotics exceeded this required therapeutic dose during the entire 2‐week storage period. From the beginning of storage, LAB maintained their viability at ≥10^9^ cfu/mL, and yeasts in group B maintained their viability at ≥10^8^ cfu/mL.

The fact that potential probiotics maintained their viability and increased, albeit at low levels, during the storage process indicated that the product produced creates an ideal environment for these microorganisms. Similar findings were obtained in the studies by Drgalic et al. (2005) and Hernandez‐Mendoza et al. (2007), and it was determined that LAB was at therapeutic doses in probiotic‐fermented drinks. Using *S. boulardii* combined with LAB in fermented dairy products significantly affected product quality and nutraceutical properties. Parrella et al. (2012) conducted research indicating that the concurrent fermentation of LAB and *S. boulardii* (CNCM I745, lyophilized form) in various milk types (raw, pasteurized, or UHT) exhibited a preference for the yeast. This preference is due to the yeast's inability to ferment or metabolize lactose. However, the yeast can utilize organic acids produced from the fermentation of glucose and galactose, promoting its growth. Simultaneously, this co‐fermentation ensures the stability of LAB strains during storage. Furthermore, these partnerships enhance the final fermented products' antioxidant properties. Consequently, the co‐growth of probiotic yeasts and LAB aids the survival of probiotic bacteria, particularly when pH levels are stabilized. It was thought that the increase in the *S. boulardii* T8‐3C counts to the therapeutic dose during the fermentation process and the preservation of both LAB and yeast viability at a level that could provide a probiotic effect during the storage process were associated with the effects stated in the study of Parrella et al. (2012). However, in contrast, Karaolis et al. (2013) found that the counts remained below the therapeutic dose in goat yogurt produced with yogurt starter culture and *S. boulardii*. The researchers have reported that this may be due to a low initial inoculation rate, a high incubation temperature, the antagonistic effect of LAB, and the inability of *S. boulardii* to utilize lactose.

### 3.3 Proteolysis levels of the groups

**FIGURE 5:**
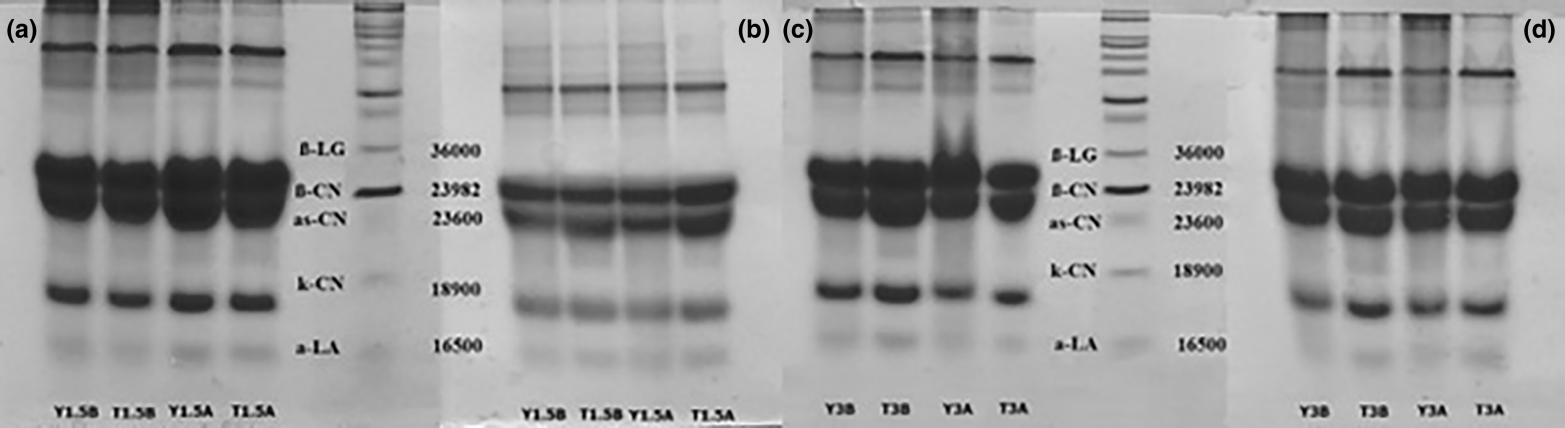
Protein profiles of probiotic‐fermented dairy drinks A; 1.5% fat day 15, B; 1.5% fat day 1, C; 3% fat day 15, D; 3% fat day 1.

The SDS‐PAGE profiles of the experimental groups yielded a differing pattern (Figure 5).

The SDS‐PAGE profile showed no differences between the replicates belonging to groups A and B. However, there was a change in the intensity of protein bands in the groups produced from milk with different storage periods and fat content. During the 15‐day storage period, proteolysis caused a significant decrease in the band intensity of serum proteins and casein fractions, especially in both groups produced from semi‐skimmed milk. The effects of proteolysis were present in the groups produced using whole milk, albeit to a lesser extent. Since the proteolysis level of B groups containing *S. boulardii* was primarily similar to that of A groups, it can be assumed that LAB is responsible for proteolysis. In various studies, proteolysis was determined in different protein fractions and levels depending on the probiotics used to produce fermented dairy drinks. Bonczar et al. (2016), in yogurt, kefir, and drinks with *Bifidobacterium bifidum* and *Lactobacillus acidophilus*, determined proteolysis levels of milk proteins. The study demonstrated that kefir exhibited the least amounts of αs‐ and β‐casein, α‐lactalbumin, and peptides in comparison to the other drinks. Conversely, it displayed the highest concentration of γ‐casein, indicating an elevated hydrolysis rate within this particular drink. Prior research has indicated that throughout the storage of fermented dairy drinks, there is a notable rise in the levels of lactoferrin, serum albumin, and peptides, accompanied by a decrease in κ‐casein content. Lá et al. (2020) found that in the probiotic‐fermented dairy drinks, they produced those different probiotics that caused proteolysis in different proteins during the storage process. In this respect, the results obtained in the present study have similarities and differences with those obtained in similar studies.

Proteolysis within dairy items typically involves the breakdown of proteins through the action of intrinsic milk enzymes and enzymes derived from LAB. This process yields peptides of moderate to low molecular weight, along with individual amino acids. The peptides generated as a result of proteolytic action on milk proteins remain inert while enclosed within the parent protein structure. Nonetheless, upon liberation, these peptides have the potential to confer advantageous health effects. These encompass actions such as inhibiting the angiotensin‐converting enzyme, as well as displaying properties like opioid, antioxidant, antidiabetic, immunomodulatory, and antimicrobial activities (Lá et al., 2020; Mora et al., 2019). In this manner, it was thought that the LAB used in the present study could produce bioactive peptides.

As mentioned above, proteolysis levels also differed in groups with different fat contents. Similarly, Fenelon et al. (2000) detected an increase in casein contents; hence, casein‐derived peptides (i.e., αs1‐CN f 1–23 and αs1‐CN f 24–199) in cheddar cheese related to the decrease in fat content during the ripening period. Therefore, more substrate for conversion to amino acid N is provided. When expressed as a percentage of total cheese, amino acid N increased significantly with decreasing fat content.

### 3.4 Sensory properties

**FIGURE 6:**
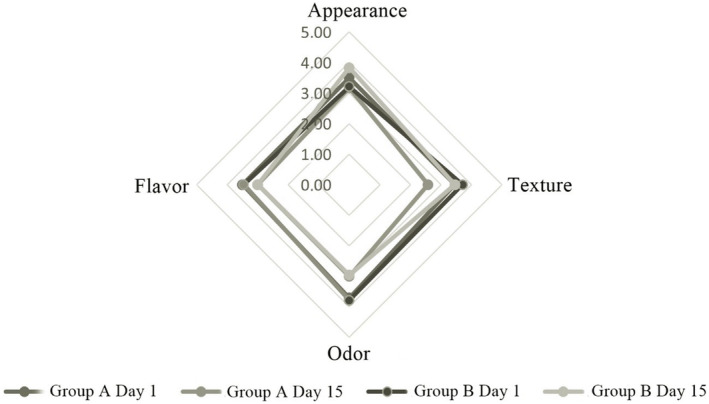
Changes in the sensory properties of group A and B probiotic fermented dairy drinks during storage.

The probiotic‐fermented dairy drinks produced in the present study were evaluated in terms of appearance, consistency, odor, taste, and general acceptability on days 1 and 15 of the storage period. The changes in these properties during storage time are shown in Figure 6.

Comparing the results of the day 1 and day 15 sensory analyses of group A and B probiotic‐fermented dairy drinks, no significant effects of storage time were observed regarding appearance and consistency. In contrast, all groups received higher scores on day 1 than day 15 regarding odor and taste properties. However, the changes in the sensory characteristics of the groups during storage were statistically not significant (*p* > .05).

Flavor and aroma are recognized as the most crucial factors influencing consumer acceptance and purchasing choices concerning dairy products (Gomes et al., 2013). In fermented dairy drinks, organic acids are the primary fermentation byproducts that significantly affect flavor. The development of acidity leads to an increase in tanginess and aroma. Across all groups, during the storage period, the augmentation of acidity coupled with a decrease in pH has been linked to an unfavorable impact on the product's taste. Moreover, alterations occurring in milk fat and proteins through fermentation also contribute to the modulation of flavor. Milk fat and peptides formed from proteolysis are essential to the flavor of fermented dairy drinks. Dairy fat plays a crucial role in the production of fermented drinks, exerting a significant influence on taste perception. The process of lipolysis leads to the liberation of unbound fatty acids. Notably, these constituents, particularly SCFA, actively participate in shaping the sensory characteristics of the end products (Beshkova et al., 1998). In the present study, no significant differences were found between the flavors of the groups with different milk fat contents (*p* > .05). It can be accepted that the flavor components produced by the proteolytic activity of the potential probiotics used in the present study were effective in improving the flavor in the groups produced with semi‐skimmed milk. It has been indicated that the excellence of fermented dairy products relies on the proteolytic efficiency of the employed strains, given that the flavor is directly influenced by the amino acids and peptides generated (Ahtesh et al., 2018). Therefore, the results of SDS‐PAGE profiling showed that proteolysis took place in the samples, and the sensory analysis results were thought to be compatible. Proteolysis can produce phenylalanine peptides that cause bitterness and flavor components consumers appreciate (Kawakami et al., 2014). The decrease in the overall acceptability of all groups may have been caused by proteolysis products that negatively affected the flavor.

### 3.5 Statistical analysis

The regression and PCA results of all analyses are given in Table 2.

**TABLE 2:** Standardized regression coefficients (SRCs) and regression coefficients (RC) showing the effects of independent variables on model predictions.

	DM***		pH*		LA*		SN**		WHC**	
	*R* ^2^ [*R* ^2^_adj._]	0.94 [0.86]	*R* ^2^ [*R* ^2^_adj._]	0.99 [0.97]	*R* ^2^ [*R* ^2^_adj._]	0.96 [0.91]	*R* ^2^ [*R* ^2^_adj._]	0.98 [0.96]	*R* ^2^ [*R* ^2^_adj._]	0.98 [0.96]
	Term	SRC [RC]	Term	SRC [RC]	Term	SRC [RC]	Term	SRC [RC]	Term	SRC [RC]
1	F	0.96 [0.67]**	MxF	−0.68 [−0.2]*	S	0.71 [0.01]^ns^	F	−0.74 [−5.52]**	F	0.74 [5.52]**
2	M	−0.21 [−0.11]^ns^	S	−0.65 [−0.01]ᶦ	M	0.67 [0.07]^ns^	M	0.7 [3.9]ᶦ	M	−0.7 [−3.9]ᶦ
3	S	−0.17 [−0.02]^ns^	M	0.31 [0.02]^ns^	MxF	−0.33 [−0.01]^ns^	S	0.37 [0.37]^ns^	S	−0.37 [−0.37]^ns^
4	MxF	0.04 [0.01]^ns^	FxS	−0.22 [0.00]^ns^	F	0.23 [0.03]^ns^	MxF	−0.56 [−1.33]ᶦ	MxF	0.56 [1.33]ᶦ
5	FxS	−0.07 [0.00]^ns^	MxS	−0.16 [0.00]^ns^	FxS	0.15 [0.00]^ns^	FxS	−0.46 [0.17]^ns^	FxS	−0.46 [−0.17]^ns^
6	MxS	0.02 [0.00]^ns^	F	0.07 [0.01]^ns^	MxS	0.04 [0.00]^ns^	MxS	0.02 [0.01]^ns^	MxS	−0.02 [−0.01]^ns^
	MC	0.00 [8.52]***	MC	0.00 [4.51]***	MC	0.00 [0.92]***	MC	0.00 [40.83]***	MC	0.00 [59.17]***

	*L*ᶦ		*a* ^ı^		b**		C*		*H*°***	
	*R* ^2^ [*R* ^2^_adj._]	0.53 [0.40]	*R* ^2^ [*R* ^2^_adj._]	–	*R* ^2^ [*R* ^2^_adj._]	0.77 [0.66]	*R* ^2^ [*R* ^2^_adj._]	0.32 [0.29]	*R* ^2^ [*R* ^2^_adj._]	0.91 [0.87]
	Term	SRC [RC]	Term	SRC [RC]	Term	SRC [RC]	Term	SRC [RC]	Term	SRC [RC]
1	FxS	−1.03 [−0.12]ᶦ	M	–	MxF	1.08 [3.62]^ns^	S	−0.57 [−0.38]**	S	−0.91 [−6.05]**
2	M	−0.97 [−1.68]ᶦ	F	–	M	−1.03 [−8.23]^ns^	M	–	MxF	−0.38 [−6.03]^ns^
3	MxF	0.81 [0.60]^ns^	S	–	*S*	0.90 [1.23]*	F	–	MxS	−0.36 [−1.41]^ns^
4	F	0.42 [0.94]^ns^	MxF	–	MxS	0.86 [0.70] ^ns^	MxF	–	M	0.26 [10.50]^ns^
5	S	0.33 [0.10]^ns^	MxS	–	FxS	−0.04 [−0.02]^ns^	MxS	–	FxS	−0.04 [−0.11]^ns^
6	MxS	–	FxS	–			FxS	–		
	MC	0.00 [22.22]***	MC	0.00 [−5.97]***	MC	0.00 [−11.02]*	MC	0.00 [11.83]***	MC	0.00 [239.96]***

*Note*: Significance Levels: *p* ≤ .0001 for ‘***’, *p* ≤ .001 for ‘**’, *p* ≤ .01 for ‘*’, *p* ≤ .05 for ‘ᶦ’, for the unimportant ‘^ns^’. “+” and “−” signs represent positive and negative relationships. Results are given as SRC [RC].

Abbreviations: *a*, green‐red color value; *b*, blue‐yellow color value; *C*, Chroma; F, Fat Content of Milk; *H*°, Hue; *L*, Lightness; M, Microorganism group; MC, Model Constant; S, Storage time (day); SN, Syneresis; WHC, Water Holding Capacity.

Since regression coefficients are expressed in different units, it is challenging to compare them directly. In some cases, the variable with the smaller regression coefficient may have a more significant effect. SRCs are easier to compare since all variables have a single unit. SRCs ascertain the extent to which a modification of one standard deviation in the independent variable is projected to bring about a corresponding alteration in the dependent variable's value by a certain number of standard deviations. For these reasons, by comparing the absolute values of the SRCs with each other, a general inference can be made about the significance of the independent variables (Siegel, 2016). The SRCs and regression coefficients of the developed regression models are shown in Table 2. Using the SRCs in these tables, the model parameters were ranked according to their importance levels, from higher to lower levels. In addition to the SRCs, regression coefficients are also available in the tables. Accordingly, regression models can also be used if desired by the reader. However, only the SRCs will be considered in the comparison of the importance of the parameters. The “±” sign of the SRCs indicates the direction of the effect of the relevant independent variable on the dependent variable. If this sign is “+,” the relationship is in the direction of direct proportion (positive), whereas if it is “−,” it is in the direction of inverse proportion (negative).

**FIGURE 7:**
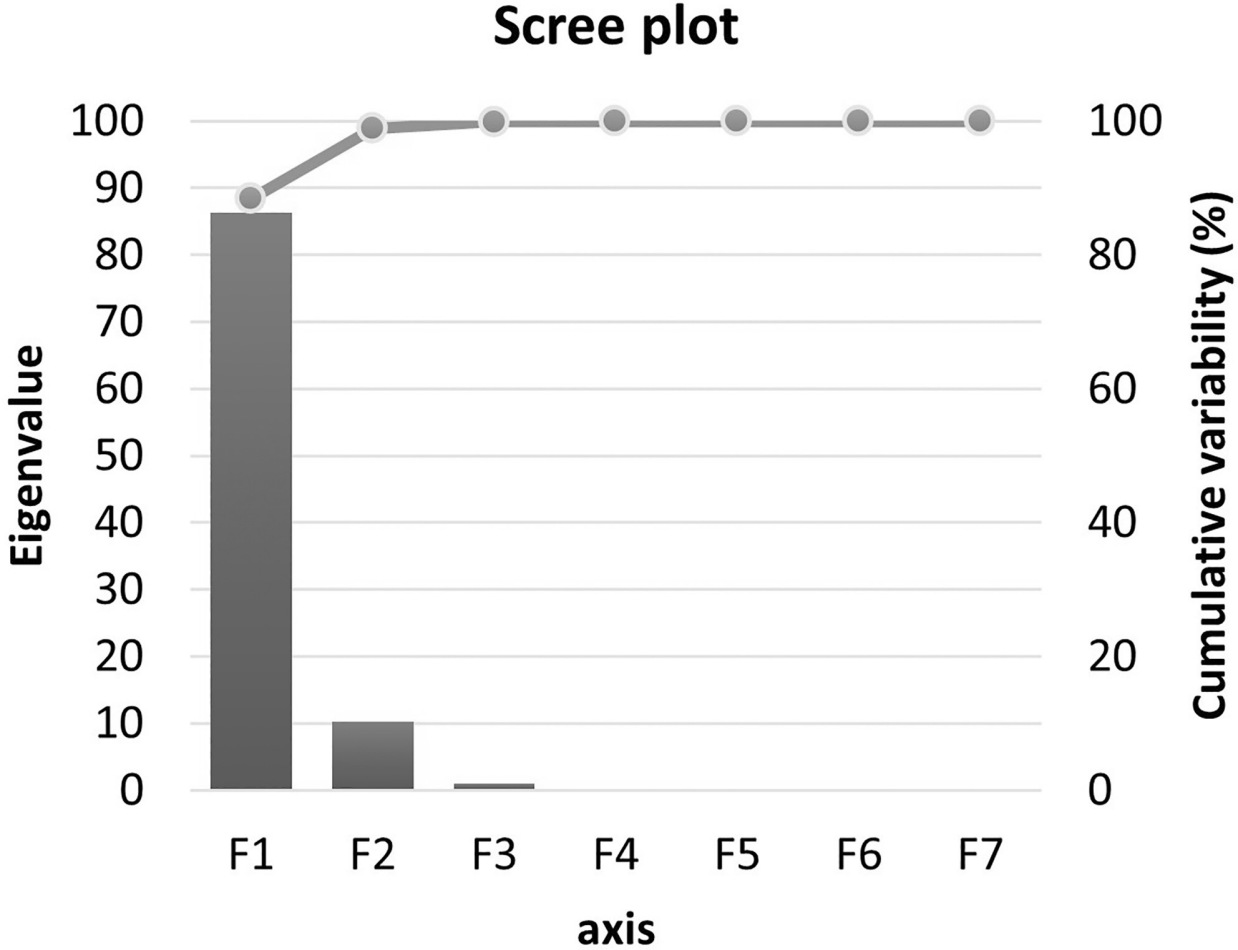
The effects of variables on total variance.

**FIGURE 8:**
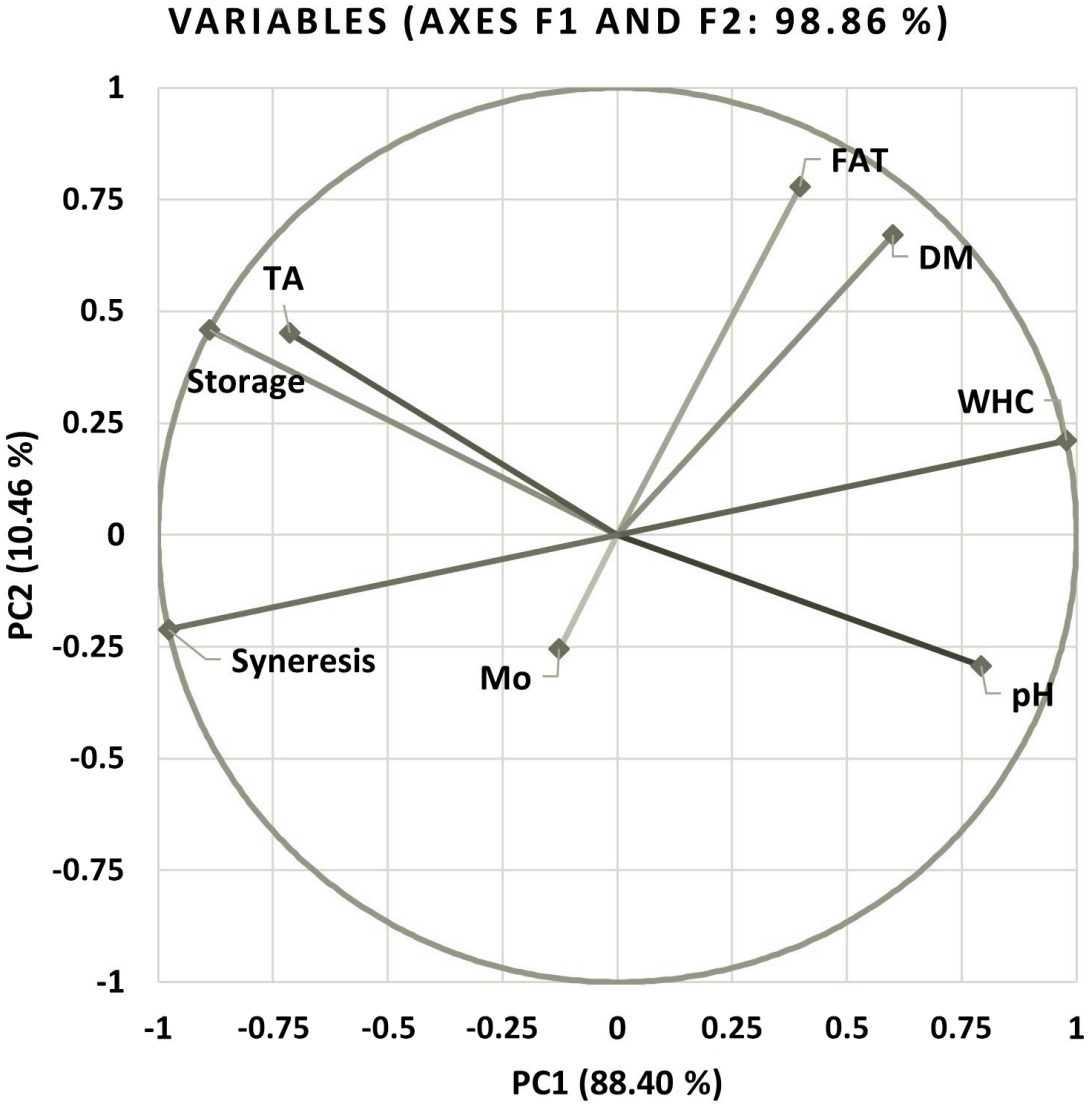
The effects of principal components on total variance. DM, dry matter; Mo, microorganism group; TA, titratable acidity; WHC, water holding capacity.

As a result of the PCA, 99.85% of the total variance can be explained by the first three principal components. The first two principal components were used to explain the relationship between the variables, with variance explanation ratios of 88.4% and 10.46%, respectively. Thus, 99.85% of the total variance can be explained by Figures 7 and 8.

Accordingly, the variables that contributed the least to the changes in the data were the microorganism group and the a* color value. Therefore, it can be stated that the microorganism group generally had no significant relationship with the other variables. Only syneresis, water holding capacity, and L* color values had a weak correlation. It can be argued that there was a positive relationship between the microorganism group and these variables.

The variables with the strongest relationship with storage time were the b* color value in the positive direction and the *C* and *H*° color values in the negative direction. In descending order, the relationships between storage time and other variables mentioned above are pH, lactic acid content, syneresis, water holding capacity, L*, and dry matter.

A strong negative correlation was found between the fat content and syneresis. A strong positive correlation exists between the fat content, water‐holding capacity, and dry matter in descending order. Shobharani and Agrawal (2009) have reported that the fat content of the probiotic‐fermented dairy drink produced using the *Leuconostoc mesenteroides* starter culture, a potential probiotic, decreased from 4.7% to 4% during the 5‐day storage period. It can be argued that the fat levels in probiotic‐fermented dairy drinks vary mainly depending on the fat content in the milk, the storage time, and the starter culture used.

## 4 CONCLUSION

Dairy products rank first among probiotic‐containing foods in the world. Therefore, the present study aimed to develop a novel probiotic‐containing fermented dairy drink suitable for consumer taste. For this purpose, potential probiotic stains isolated in previous studies were used. The changes in the storage process of products produced with two different probiotic combinations from whole and semi‐skimmed milk were examined to meet consumer expectations. The probiotic content required at least 1 × 10^6^/mL in the products was provided; this value was 8.56 log cfu/mL on day 15 of the storage. The physicochemical and microbiological properties of the products did not show any significant changes during storage. Also, all groups were found acceptable regarding sensory properties on days 1 and 15. With these characteristics, a fermented dairy drink containing probiotics can be considered a new commercial product.

## AUTHOR CONTRIBUTIONS

**Oğuzhan Gedik:** Formal analysis (equal); writing – original draft (supporting). **Aynur Gül Karahan:** Conceptualization (lead); formal analysis (equal); writing – original draft (lead).

## CONFLICT OF INTEREST STATEMENT

The authors declare no conflict of interest.

## Data Availability

Data will be made available on reasonable request.
